# Can coffee or chewing gum decrease transit times in Colon capsule endoscopy? A randomized controlled trial

**DOI:** 10.1186/s12876-018-0824-9

**Published:** 2018-06-25

**Authors:** Maria Magdalena Buijs, Morten Kobaek-Larsen, Lasse Kaalby, Gunnar Baatrup

**Affiliations:** 1 Department of Clinical Research, University of Southern Denmark, Winsløwsparken 19, 3rd floor, 5000 Odense, Denmark; 2 Department of Surgery, Odense University Hospital, Baagøes Allé 15, Forskningshus, 5700 Svendborg, Denmark

**Keywords:** Colon capsule endoscopy, Transit time, Excretion rate, Bowel preparation, Coffee, Chewing gum, Bowel cleansing

## Abstract

**Background:**

A high rate of complete colon capsule endoscopy (CCE) investigations is required for a more widespread use of CCE. The objective of this study was to assess if coffee or chewing gum can increase excretion of the colon capsule within battery life time (excretion rate).

**Methods:**

One hundred eighty six screening participants with a positive immunochemical fecal occult blood test were included in this single-centre randomized controlled trial with blinding of the investigators to the randomization. Participants received instant coffee, chewing gum or nothing in addition to the standard bowel preparation.

**Results:**

The intention was to include 57 participants in the coffee group, 61 in the chewing gum group and 60 in the control group, on 8 participants data were missing. A total of 165 participants were included in a per protocol analysis. Exclusion was due to not receiving the allocated intervention (8 coffee, 4 chewing gum) and technical failure of the capsule (1 coffee). The excretion rate was 58% in the coffee group (*n* = 48), 63% in the chewing gum group (*n* = 57) and 55% in the control group (*n* = 60, *p >* 0.2). Transit times were similar in all groups. The excretion rate was low in participants who had transit times over 10 h (14%). A strong correlation was found between adequate cleansing and excretion of the capsule.

There were no serious adverse events related to the interventions or CCE investigations.

**Conclusions:**

Chewing gum and coffee did not improve excretion rate in this study. An effect of chewing gum could not be proven, possibly due to sample size. Since chewing gum might improve excretion rates, is cheap and has no known side effects, it needs to be considered in future bowel preparation trials for CCE.

**Trial registration:**

NCT02303756, registered on December 1st 2014.

**Electronic supplementary material:**

The online version of this article (10.1186/s12876-018-0824-9) contains supplementary material, which is available to authorized users.

## Background

Colon capsule endoscopy (CCE) was introduced in 2006 as a new imaging technique of the colon [1]. CCE does not require sedation or analgesia and the patient can stay at home during the investigation. The European Society of Gastrointestinal Endoscopy has approved the use of CCE in average risk patients, after an incomplete colonoscopy and in patients who refuse or have contraindications for a colonoscopy [2]. In recent years CCE has been studied in colorectal cancer screening participants with promising results [3–5]. CCE might improve the screening program by selecting participants with proven neoplasia for therapeutic colonoscopy, since the low sensitivity of the immunochemical fecal occult blood test (iFOBT) induces a substantial number of false negative results [6]. Limiting factors for the use of CCE are the cleanliness of the colon during the investigation and the number of capsules that are not excreted within battery life time [1]. In a recent meta-analysis a completion rate of 90.5% (95% CI: 88.3–92.4%) was calculated for CCE, which is similar to the rate of complete screening colonoscopies in Denmark of 89% [7, 8]. Multiple studies have assessed bowel preparation and boosters for CCE in order to enhance cleanliness and reduce transit times [9–14]. According to Danish guidelines all screening participants should receive a standard polyethylene glycol-electrolyte (PEG) based bowel preparation, which leads to low excretion rates. The use of boosters other than PEG was not allowed and therefore nonmedical interventions were considered as an alternative to improve completion rates. Coffee and chewing gum have been reported to reduce transit time in small bowel capsule endoscopy and after abdominal surgery [15–17]. Some clinics already use coffee or chewing gum as part of their CCE bowel preparation, but their effect has not been studied in CCE as yet. The objective of this study was to investigate if addition of either coffee or chewing gum to the standard CCE bowel preparation can increase excretion during battery life time.

## Methods

### Study design

This study was a sub study within the Care for Colon study in which home delivered CCE was investigated in iFOBT positive screening participants (ClinicalTrialst.gov: NCT02303756) [18]. This study was conducted as a single-center randomized controlled trial, with blinding of the investigators to the randomization. Randomization in three equal groups was achieved by closed envelops with allocated interventions, which were opened by an independent research nurse prior to the ingestion of the colon capsule. Participants had the possibility to decline the intervention, which lead to exclusion from analysis. The primary endpoint was the excretion rate of CCE in the different intervention groups. Secondary endpoints were total transit time, colon transit time, the number of capsules retained before the cecum and bowel cleansing quality in the different intervention groups.

### Study population

Participants of the Care for Colon study were recruited from the national screening program on the island of Funen, Denmark, which includes average risk individuals between 50 and 74 years of age. In case of a positive iFOBT they were invited to participate in the trial and were included after obtaining informed consent. Exclusion criteria included previous bowel surgery (except for appendectomy), inflammatory bowel disease, ostomy and clinical symptoms of bowel obstruction in the last three months, diabetes mellitus and vomiting during the preparation.

### Interventions

All groups received standard bowel preparation with PEG (Moviprep®) and PEG boosters after ingestion of the colon capsule, as described in the Additional file 1: Table S1. All participants were assisted with ingestion of the colon capsule by a study nurse. The intervention was timed based on an alarm that went off when the capsule left the stomach. The coffee group was asked to drink one cup of instant coffee (57 mg caffeine) three hours after the capsule left the stomach. The chewing gum group chewed two pieces of sugar free chewing gum to chew for at least 30 min after the capsule left the stomach. The control group did not receive any intervention.

### CCE analysis

All CCE investigations were performed with a second generation CCE (PillCam2®, Given Imaging, Israel). The CCE images were analyzed by experienced gastroenterologists who selected the first cecum image and last (rectal) image, which contained time stamps corresponding to the time since intake of the capsule. Bowel cleansing quality was assessed by the gastroenterologist based on the Leighton-Rex scale as either adequate or inadequate [19].

### Data collection

Basic characteristics including sex and age were collected prospectively. Data on the capsule as excretion of the capsule, capsules retained before the cecum, total transit time, colon transit times and bowel cleansing quality were collected prospectively from the CCE evaluation reports.

### Sample size calculation and statistics

This study was designed with the assumption that the best intervention would yield an excretion rate of 90%, the next 75% and the last group 60%. Based on this assumption and a power of 90%, a total of 49 participants should be included in each group. Adjusting for a drop-out rate of 10% each group needed to include 54 participants. We decided on including 60 participants in each of the three groups.

The results were analyzed by a per protocol analysis, in order to reliably assess the effect of the interventions [20]. Basic characteristics are presented as means with standard deviations for continuous variables and percentages for binary variables. Transit times are presented as medians with range, after proving absence of normality with the Shapiro-Wilk test. Significance was tested using Chi^2^ test and Fishers Exact Test for binary variables and Kruksal Wallis test for non-normal distributed continuous variables. In addition, a univariate and multivariate regression analysis on variables effecting excretion rate was conducted. Statistical analyses were performed using Stata IC 15.0.

## Results

A total of 186 participants were included in the study from October 2015 to July 2016. Due to missing data on allocation 8 participants were excluded from analysis. The intention was to treat 57 participants with coffee, 61 with chewing gum and include 60 control participants. All 12 participants that refused their allocation (8 coffee and 4 chewing gum) were excluded from the per protocol analysis. In the coffee group one study was incomplete due to technical failure of the colon capsule three minutes after ingestion and therefore excluded from per protocol analysis.

**Fig. 1:**
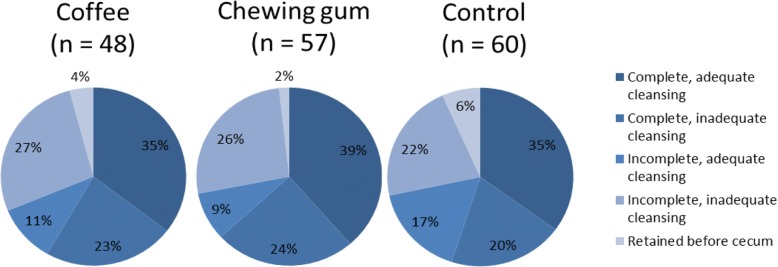
Excretion and bowel cleansing quality by intervention

**Fig. 2:**
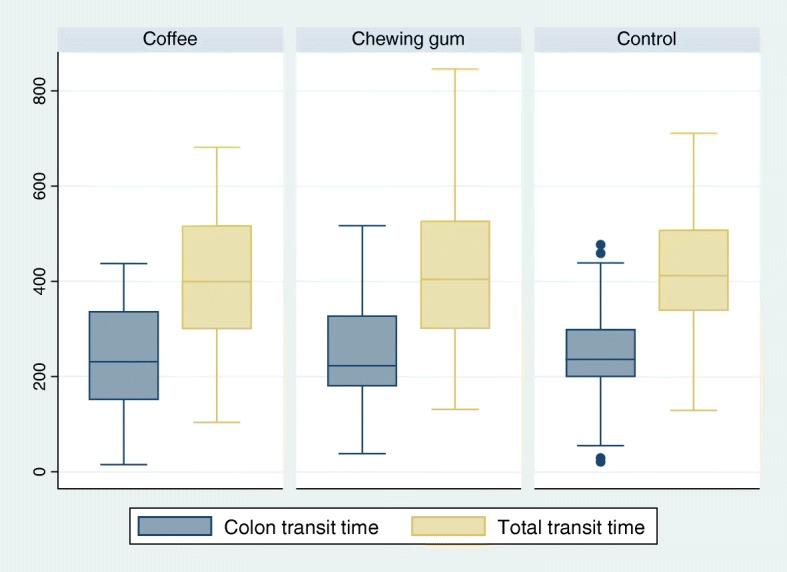
Colon and total transit time in complete CCE investigations by intervention Transit times in minutes presented as a boxplot with median, quartiles and outliers (dots).

Sixty-six % (*n* = 109) of participants were male and their average age was 64 ± 7 years. The age of participants was similar in all groups, but there was a trend towards more male participants in the chewing group (77% vs 55 and 63% in respectively the coffee and control group, *p* = 0.052). The excretion rates, bowel quality and amount of capsules that was retained before the cecum are displayed in Fig. 1. There were no significant differences between the groups, even though the chewing gum had the highest excretion rate of 63% (95% CI: 49–76) in comparison to coffee (58%; 95% CI: 43–72) and controls (55%; 95% CI: 42–68). Chewing gum had also the lowest number of capsules retained before the cecum. Colon and total transit times were similar in all groups (Fig. 2). Previous studies generally presented the total transit time in categories, which are presented in Additional file 1: Figure S1.

A sub analysis was performed to analyze possible factors that influenced excretion rate (Table 1).

**Table 1:** Univariate analysis on excretion of capsules

	Non-excreted (*n* = 68)	Excreted (*n* = 97)	*P*
Age (years)	66.0 (64.3–67.6)	62.7 (61.2–64.2)	*< 0.01*
Sex (male)	62% (49–73)	69% (59–78)	> 0.2
Bowel cleansing (adequate)	30% (19–42)	62% (51–72)	*< 0.001*

All variables are presented with 95% confidence intervals

Both young age and an adequate bowel cleansing were correlated to improved excretion rates, but in a multivariate analysis none of these factors significantly influenced excretion.

Technical failures did not seem to influence battery life time, since the average total transit time in the incomplete group was almost 15 h. The two participants with an incomplete investigation and a transit time of less than 10 h; respectively 6.5 and 9.5 h did have blackouts during the video. The majority of participants with a transit time of less than 10 h had a complete investigation, contrary to the group with a longer transit time (98% vs 14%).

There were no serious adverse events related to the interventions or CCE investigations.

## Discussion

In order to avoid unnecessary diagnostic colonoscopies by the use of CCE, a high excretion rate is essential. In this randomized controlled trial addition of chewing gum to the standard protocol improved excretion rates and decreased the number of capsules that were retained before the cecum. This effect was not significant, which might be due to a type II error. The effect of coffee was inferior to that of chewing gum in this study. A sub analysis showed that a younger age and adequate bowel cleansing quality had a strong correlation with excretion of the capsule within battery life time, but a causal effect could not be demonstrated.

One study reported an excretion rate of 76% with a similar preparation asused in this study [11]. Our excretion rate of 58% might be explained by differences in the protocols: 1) bowel preparation in the study by Hartmann et al. was started one day instead of two days prior to the investigation; 2) PEG boosters were administered two and 6 h after ingestion of the capsule instead of dependent on the moment the capsule left the stomach; 3) Hartmann et al. performed a pilot study including 49 patients; and 4) our study was performed as an out clinic instead of in clinic procedure.

Methodologic limitations of this study are the exclusion of participants after randomization and unclear allocation of intervention in eight objects, which caused the coffee group to be smaller than the other groups; and the absence of a sample size calculation. The sample size of this study was not powered to prove a difference of 8%, therefore a significant effect cannot be demonstrated. This study might lack effect because the interventions were applied only once, whereas previous studies distributed the intervention multiple times [15–17, 21]. Additionally, the interventions were dependent on passage through the stomach, instead of planned on set times after ingestion of the capsule.

The exact physiologic effects of coffee and chewing gum on the bowel are still unknown. A study in healthy volunteers indicates that coffee increases the rectosigmoid motility in about a third of the individuals [22]. Coffee has been shown to reduce time to first bowel movement after colorectal and gynecologic surgery by 9.4–15.4 h on average [17, 23, 24]. A recent meta-analysis on the effect of chewing gum on postoperative ileus after colorectal surgery, including 1736 patients from 18 RCTs, observed a reduction of 16.4 h (95% confidence interval: − 22.7; − 10.2) of time to first bowel movement [25]. One of the two studies in small bowel capsule endoscopy found a significant reduction of transit time. However this study was conducted as a pilot study without a sample size calculation [15]. Both studies distributed chewing gum immediately after ingestion of the capsule and every 2 h afterwards. If coffee and chewing gum would have been applied immediately after ingestion of the capsule and repeated every 2–3 h afterwards, the effect of these interventions might have been stronger than demonstrated in this study.

## Conclusions

Increasing the rate of complete CCE investigations is necessary for the implementation of CCE in clinical routine diagnostics. Chewing gum did improve the excretion rate in this study (not significantly), is cheap and has no known side effects; therefore the addition of chewing gum should be considered in future bowel preparation studies. The strong correlation between adequate bowel cleansing quality and complete investigations, suggests that an effective bowel preparation will improve both cleansing quality and completion rate and inspires to continue investigating bowel preparations for CCE.

## Additional file

Additional file 1: **Table S1.** Bowel preparation with timing of PEG cleansing and boosters. **Figure S1.** Total transit time in categories of CCE investigations by intervention. (DOCX 31 kb).

## Abbreviations

CCE: Colon Capsule Endoscopy
iFOBT: Immunochemical fecal occult blood test
PEG: Polyethylene glycol-electrolyte
