# Supplementary material for: Can coffee or chewing gum decrease transit times in Colon capsule endoscopy? A randomized controlled trial
Source: BMC Gastroenterol. 2018 Jun 25;18:95. doi: 10.1186/s12876-018-0824-9 (PMC6020226; doi:10.1186/s12876-018-0824-9)
Supplement: Supplementary file 1 — Table S1. Bowel preparation with timing of PEG cleansing and boosters. Figure S1. Total transit time in categories of CCE investigations by intervention. (DOCX 31 kb). [file 12876_2018_824_MOESM1_ESM.docx]

**Supplementary Table 1. Bowel preparation with timing of PEG cleansing and boosters**

| **Day -2**  All day | 2 L water in addition to normal intake  2 times daily 1000 mg magnesiumoxide (oral) |
| --- | --- |
| **Day -1**  All day  Until 16.00  17.00-19.00 | Clear liquids only  White pasta with oil, only  1 L Moviprep® followed by 1.5 L water |
| **Exam day**  6.00-7.30  7.30-9.30 | 1 L Moviprep ® followed by at least 1.5 L water  No food/liquid ingestion |
| ± 10.00 | Ingestion of CCE with 20 mg domperidon (oral) |
| After alarm^¶^ | 0.75 L Moviprep ® followed by at least 0.6 L water |
| + 3 hours | 0.25 L Moviprep ® followed by 0.2 L water |
| + 2 hours | Bisacodyl enema |

^¶^The CCE-belt gave an alarm when the capsule left the stomach.

**Supplemerentary Figure 1. Total transit time in categories of CCE investigations by intervention**
